# Climate Change, Urbanization, and the Emerging Urban Threat of Rift Valley Fever in Tropical and Subtropical Cities: A Narrative Review

**DOI:** 10.3390/tropicalmed11080226

**Published:** 2026-08-12

**Authors:** Ahmad Y. Alqassim

**Affiliations:** Department of Family & Community Medicine, College of Medicine, Jazan University, Jazan 45142, Saudi Arabia; aalqassim@jazanu.edu.sa

**Keywords:** Rift Valley fever, urbanization, climate change, *Culex quinquefasciatus*, peri-urban, One Health, inter-epidemic transmission, vector-borne zoonosis

## Abstract

Rift Valley fever (RVF) is a climate-sensitive mosquito-borne zoonosis long regarded as a rural, pastoral disease, yet accelerating tropical urbanization and intensifying climate variability may be reshaping its epidemiology at the urban–peri-urban interface. This narrative review examines how global climate change and urban-specific climatic conditions jointly shape RVF virus (RVFV) vector habitats, transmission, and burden in tropical and subtropical cities, synthesizing 51 of 412 English-language records identified by a structured, non-systematic search of PubMed, Scopus, Web of Science, and Google Scholar (2009–2026) and selected for relevance to urban and peri-urban RVF. This research draws on human, livestock, and vector evidence from Sub-Saharan Africa, the Arabian Peninsula, and Indian Ocean islands across epidemic and inter-epidemic periods. The synthesis indicates that impervious surfaces, poor drainage, and open water storage can recreate the water-retaining function of rural dambos, sustaining a year-round larval habitat, and that *Culex quinquefasciatus* dominance together with peri-urban cattle may form an amplification bridge to humans. Direct evidence remains scarce, anchored by a single peri-urban serosurvey and limited urban slaughterhouse entomology. Critical gaps include urban primary-vector ecology, infection-rate data, urban-heat effects, city-specific exposure studies, and coupled climate–urban burden models. We conclude that RVF is a plausible emerging urban threat warranting proactive inter-epidemic surveillance and integration of RVF into urban planning and One Health systems.

## 1. Introduction

Rift Valley fever (RVF) is a mosquito-borne viral zoonosis caused by Rift Valley fever virus (RVFV; family *Phenuiviridae*, genus *Phlebovirus*). It is listed as a priority pathogen by the WHO, FAO, and CDC because it can cause hemorrhagic fever, encephalitis, and retinitis in humans, trigger abortion storms and neonatal death in livestock, and spread through infectious aerosols during epizootics [1,2]. Between 1999 and 2021, RVFV was reported in 39 countries, with ≥4353 confirmed human cases and 755 deaths—likely underestimates given subclinical, frequently missed infection [1,3]—alongside major economic losses, such as the ~US$471 million (≈5% of GDP) borne by Somalia in 2006–2007 [4,5]. Despite RVFV’s inclusion on the WHO R&D Blueprint, no licenced human vaccine exists, and surveillance in endemic regions remains fragmented and largely reactive [6,7]. Together, this combination of severe disease, weak surveillance, and missing countermeasures motivates the present review, which places RVF within a critically understudied context: tropical and subtropical urbanization under accelerating climate change.

Our understanding of RVF epidemiology has been shaped almost entirely by research in rural pastoral and agro-pastoral settings. In these landscapes, flooding-driven vector emergence, high livestock densities, and close human–animal contact define the main transmission pathways [8,9]. This model increasingly fails to reflect the reality of rapidly urbanizing tropical regions. The population of Sub-Saharan African cities is projected to triple by 2050, driven largely by unplanned peri-urban sprawl into flood-prone, ecologically vulnerable zones [10]. These transitional areas combine several conditions plausibly relevant to RVFV amplification: impervious surfaces that generate runoff, poor drainage, open household water storage, peri-urban livestock keeping, and proximity to live animal markets and informal slaughterhouses [10,11]. Against this backdrop, a previous study stands as the only published account of RVFV activity in a peri-urban setting. It documented inter-epidemic livestock circulation and measurable zoonotic spillover risk in northern Tanzania [12]. That this remains the sole such report underscores the systematic neglect of urban RVF and defines the central gap this review seeks to address.

**Figure 1:**
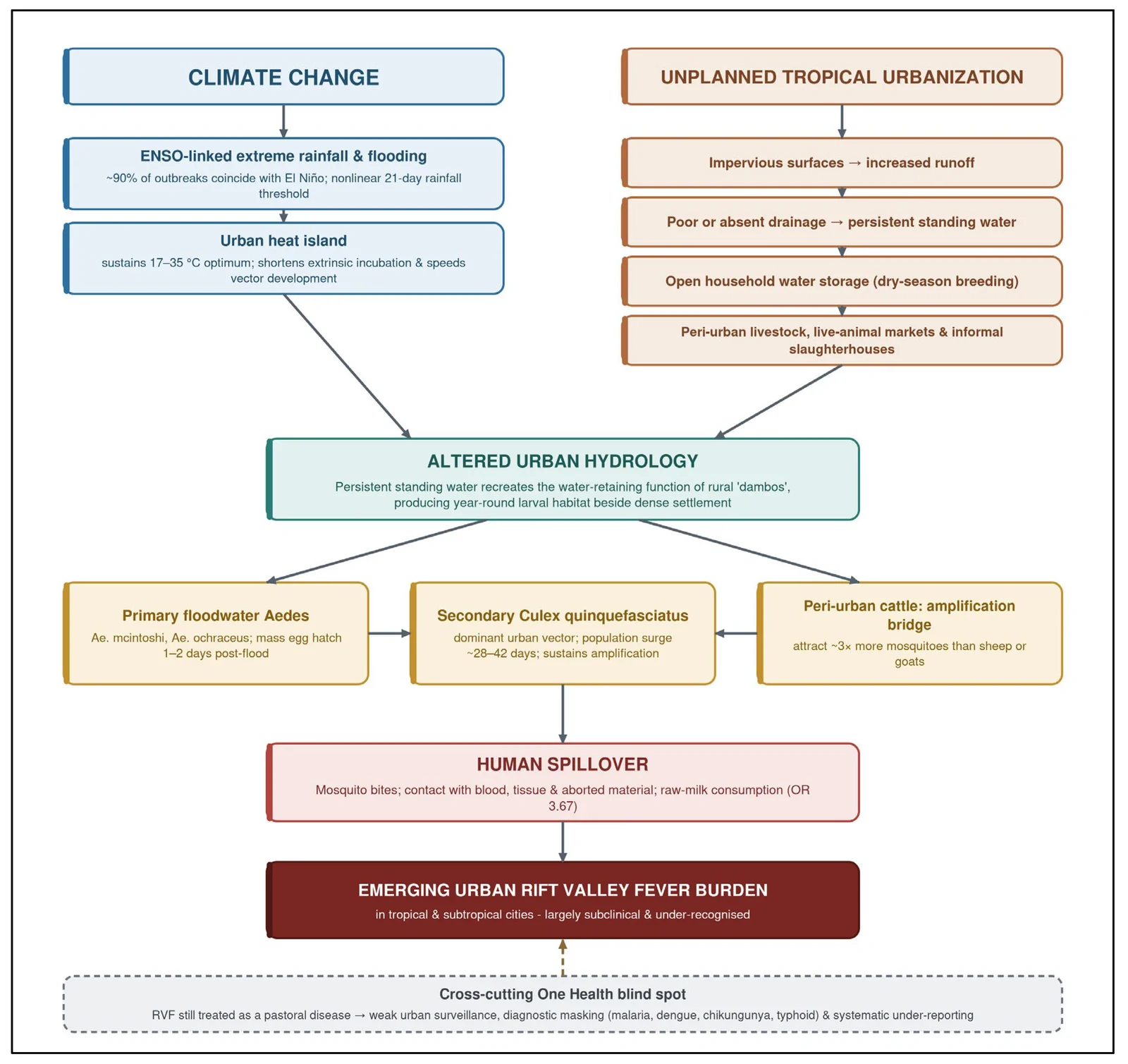
Conceptual framework linking two macro-forces; climate change and unplanned tropical urbanization; to an emerging urban Rift Valley fever (RVF) burden. This figure is an original figure created by the author using Microsoft Word for Mac (version 16.111; Microsoft Corporation, Redmond, WA, USA); its visual presentation was refined with Claude Sonnet 5 (Anthropic, San Francisco, CA, USA), without altering any data, labels, or relationships.

The neglect of urban RVF cannot be separated from two converging macro-level forces that are jointly reshaping disease risk in tropical settings. First, climate change is increasing the frequency and intensity of extreme rainfall and flooding across East Africa and the Arabian Peninsula. These are the ecological triggers that initiate RVFV outbreak cycles, prompting mass hatching of dormant, virus-infected Aedes eggs and the subsequent buildup of secondary Culex vector populations [8,13]. Second, rapid and largely unplanned tropical urbanization is pushing human settlements into historically endemic ecological zones. At the same time, it generates the infrastructure deficits (poor drainage, open water storage, and accumulating solid waste) that sustain year-round vector breeding [10,12]. Adding to these, the urban heat island may hold temperatures in the optimal range for mosquito reproduction and viral incubation year-round—well characterized for dengue vectors but unstudied for RVFV [11]. Critically, it is their interaction (mediated through altered urban hydrology, disrupted vector ecology, and novel human–livestock interfaces) that forms the subject of this review, which examines how global and urban-specific climate dynamics jointly shape the RVF burden in tropical and subtropical cities. The conceptual framework underpinning this review is summarized in Figure 1.

This narrative review pursues five linked aims: how climate change and urban-specific conditions reshape RVFV vector habitats and transmission seasonality; the evidence for urban and peri-urban circulation across vectors, livestock, and humans (epidemic vs. inter-epidemic); the socio-demographic and land-use drivers of urban risk; the projected future burden and highest-risk cities and populations; and mitigation and adaptation strategies (early warning, integrated vector management, livestock vaccination, One Health surveillance, and urban planning). Drawing on Sub-Saharan African, Arabian Peninsula, and Indian Ocean evidence across human, livestock, and vector studies, this study adds a One Health perspective to urban arbovirology and closes with critical knowledge gaps and a prioritized research and policy agenda.

Climate change intensifies ENSO-linked rainfall/flooding (~90% of outbreaks coincide with El Niño) and, via the urban heat island, sustains the 17–35 °C window favouring vectors; urbanization adds impervious surfaces, poor drainage, open water storage, and peri-urban livestock, markets, and slaughterhouses. Both converge on altered urban hydrology, where standing water recreates the function of rural dambos to sustain a year-round larval habitat. This drives amplification from primary floodwater Aedes (hatch 1–2 days post-flood) to the secondary urban vector *Culex quinquefasciatus* (surge ~28–42 days), bridged by peri-urban cattle (~3× more attractive than small ruminants), producing human spillover via bites and contact with blood, tissue, aborted material, and raw milk (OR 3.67). A cross-cutting One Health blind spot weakens surveillance, masks diagnosis, and drives under-reporting. Solid arrows represent hypothesized causal pathways; dashed arrows represent a cross-cutting modifier.

## 2. Methods

This narrative review followed a structured but non-systematic search strategy designed to capture the sparse, interdisciplinary literature on urban Rift Valley fever. PubMed, Scopus, Web of Science, and Google Scholar were searched without a start-date restriction and updated through (2009–2026), with the included literature spanning 2009 to 2026, combining terms for the pathogen and disease (“Rift Valley fever”, “RVFV”, “phlebovirus”) with terms for vector ecology (“Aedes”, “Culex”, “mosquito”, “vector competence”), climate (“rainfall”, “flooding”, “El Niño”, “climate change”, “temperature”), and urbanization (“urban”, “peri-urban”, “city”, “land use”, “livestock”, “slaughterhouse”). Reference lists of key reviews and included articles were hand-searched to identify additional sources. Priority was given to peer-reviewed studies reporting RVFV activity in vectors, livestock, or humans, alongside modelling, remote sensing, and socio-economic analyses relevant to transmission in tropical and subtropical settings; authoritative grey literature from the WHO, FAO, and United Nations was included where it supplied epidemiological, forecasting, or demographic data unavailable elsewhere. After de-duplication, 412 records were screened by title and abstract against these criteria, and 51 were retained for synthesis. Given the near absence of dedicated urban RVF studies, evidence from rural pastoral systems and from analogous urban arboviruses, principally dengue, was deliberately drawn upon to reason about plausible mechanisms by analogy. Records were restricted to English-language publications because English is the predominant language of the indexed international literature on RVF and related arboviruses, and reliable translation resources were unavailable, with geographic emphasis on Sub-Saharan Africa, the Arabian Peninsula, and the Indian Ocean islands, spanning both epidemic and inter-epidemic periods. Rather than pooling data quantitatively, the evidence was synthesized narratively and organized thematically around the review’s five objectives: climate and vector habitats, documented urban circulation, socio-demographic and land-use drivers, projected future burden, and mitigation strategies. A narrative rather than systematic approach was chosen because the urban RVF evidence base is too fragmented and heterogeneous to support meta-analysis and because this framework better accommodates the cross-disciplinary integration the topic demands.

## 3. Biology and Transmission Ecology

RVFV (order *Bunyavirales*; family *Phenuiviridae*; genus *Phlebovirus*) has a single-stranded RNA genome divided into three segments: large (L), medium (M), and small (S). The S segment uses an ambisense strategy to encode both the nucleoprotein N and the non-structural protein NSs [4,8]. The M segment encodes the glycoproteins Gn and Gc, which allow the virus to enter host cells and carry its main neutralizing epitopes. NSs is the principal virulence factor because it suppresses interferon-β production and disables the innate immune response [4,14]. Most human infections resolve as a self-limiting febrile illness. However, about 1–2% progress to severe hepatitis, retinitis, encephalitis, or hemorrhagic fever, and the pooled case fatality rate reaches 27.5% among laboratory-confirmed cases [3,7]. The disease is far more severe in livestock, where RVFV causes near-universal abortion in pregnant ewes, near-total death in newborn lambs, and prolonged high-level viremia in cattle that drives epizootic amplification [2,4]. Humans become infected through mosquito bites or through contact with the blood, tissues, aborted materials, or raw milk of viremic animals. No human-to-human transmission has been documented [5,12]. Laboratory confirmation combines direct and serologic testing: in the acute viraemic phase, real-time RT-PCR, antigen-detection ELISA, or virus isolation give direct diagnosis, while serology stages infection—ELISA-detected IgM marks recent and IgG past exposure, with the virus-neutralization test as a reference standard [15]. This acute-versus-past distinction underpins the inter-epidemic serosurveys and sentinel-herd monitoring discussed later.

**Figure 2:**
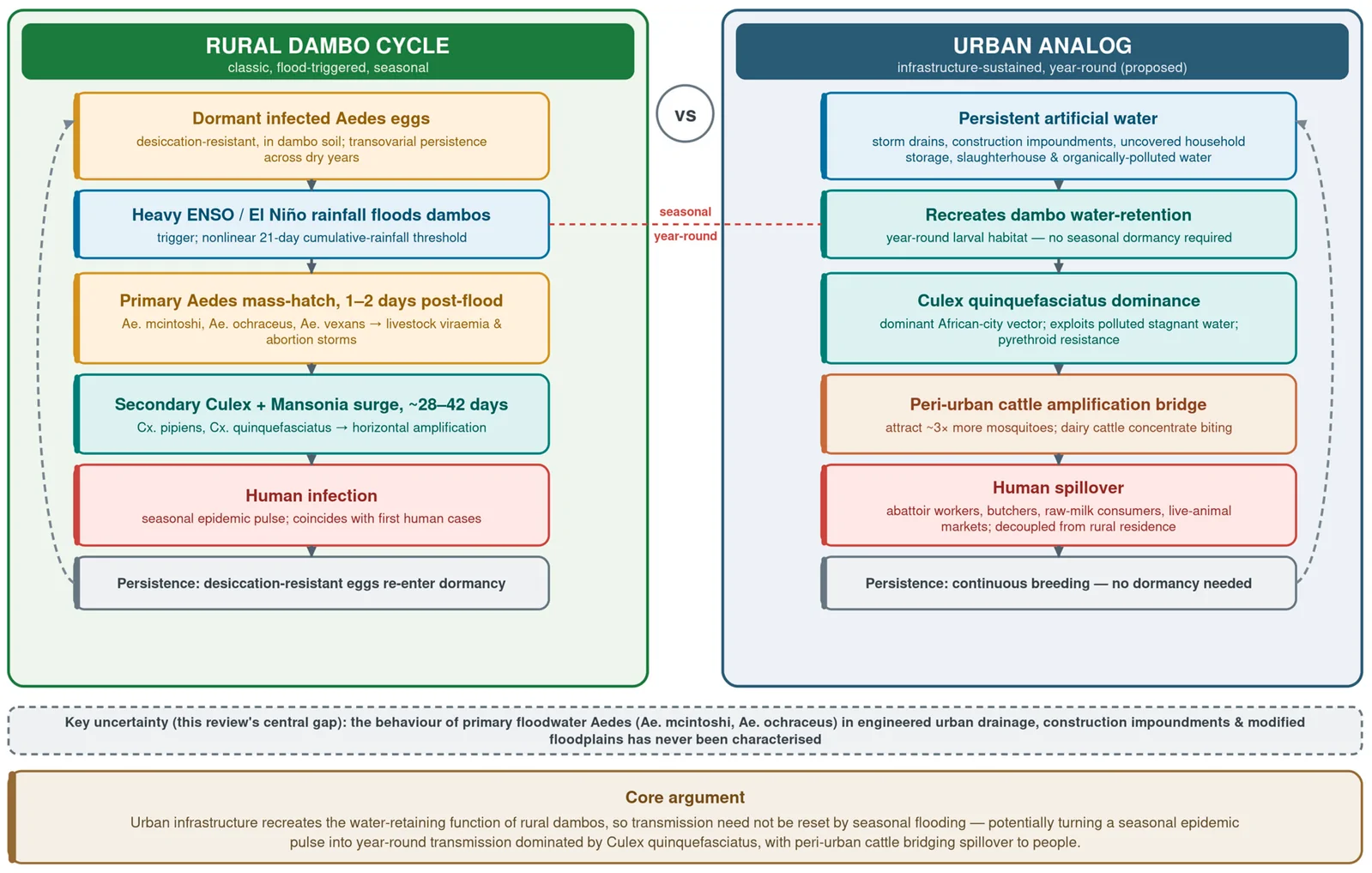
Side-by-side comparison of the Rift Valley fever virus (RVFV) transmission cycle in its classic rural setting and the urban analogue proposed by this review; equivalent stages share colour. This figure is an original figure created by the author using Microsoft Word for Mac (version 16.111; Microsoft Corporation, Redmond, WA, USA); its visual presentation was refined with Claude Sonnet 5 (Anthropic, San Francisco, CA, USA), without altering any data, labels, or relationships.

These pathogen–host dynamics play out within two ecologically distinct but connected transmission cycles. During inter-epizootic periods, RVFV persists by passing from infected female mosquitoes to their eggs (transovarial inheritance). These desiccation-resistant floodwater *Aedes* eggs lie dormant in dambo soils through successive dry years, with only limited amplification in local vertebrate hosts [8,16]. Epizootic conditions arise when unusually prolonged rainfall floods these grasslands. Infected eggs of *Aedes* mcintoshi, *Ae. ochraceus*, and *Ae. vexans* hatch en masse within one to two days of flooding, releasing infected primary vectors that quickly start livestock transmission and generate the high viremias needed for amplification [8,17]. As standing floodwater accumulates, secondary vectors (*Culex pipiens*, *Cx. quinquefasciatus*, and *Mansonia* spp.) colonize these habitats and come to dominate mosquito populations about 30–40 days after flooding, sustaining widespread horizontal transmission to humans [8]. Importantly, this secondary emergence coincides with the first human cases and marks the point at which the window for effective primary vector control has already closed [8,17]. These two ecologically linked cycles, and their hypothesized urban parallel, are contrasted in Figure 2. Across both cycles, RVFV has been isolated from more than 53 mosquito species spanning eight genera [8].

Building on this transmission ecology, RVFV has steadily expanded its geographic range since it was first described in Kenya in 1930 [4,9]. After recurrent epizootics in eastern and southern Africa, the virus reached North Africa in 1977, causing Egypt’s epidemic of an estimated 200,000 human infections and 598 deaths. It then crossed continental boundaries into Saudi Arabia and Yemen in 2000–2001, a spread driven mainly by transboundary livestock trade [5,9]. A systematic review covering 1999–2021 documented RVFV activity in 39 countries, with the highest and most recurrent burden concentrated in East Africa (Kenya, Sudan; 43% of transmission events), Madagascar, Mauritania, and South Africa [1]. Pooled seroprevalence estimates of 7.8% in humans and 9.3% in animals reveal extensive subclinical circulation that official case counts substantially underestimate [3]. The risk of further global spread is reinforced by confirmed vector competence in *Culex pipiens* and *Aedes albopictus*, both widely distributed across Europe and Asia [16], and by RVFV’s designation as a CDC Category A bioterrorism agent [2,8].

At the molecular level, RVFV circulates as multiple, geographically structured lineages (A–O): lineage C predominates in eastern Africa, lineage H in southern Africa, and lineage A in northern Africa [18]. Retrospective genomic and phylodynamic analysis places the most recent common ancestor at approximately 1918 and traces the dominant lineage C to mid-1970s Zimbabwe, from which it was independently introduced into Kenya (1983), Madagascar (1990s), and the Arabian Peninsula (2000s) and dispersed rapidly (diffusion coefficient >50,000 km^2^/year), with continued diversification and cryptic circulation during inter-epidemic periods [18]. Recent activity confirms this ongoing expansion: outbreaks have reached previously less-affected areas such as Burundi (2018) and Rwanda (2018 and 2022) [18], and in 2018 a peri-urban outbreak among dairy cattle and hospitalized febrile patients in Moshi, northern Tanzania, produced genomically confirmed, and fatal, human infection in an urban-adjacent setting during an otherwise inter-epidemic period [19]. Notably, this genomic evidence explicitly identifies urban centres among the settings where climate variability and land-use change may raise future risk [18].

In the rural cycle, desiccation-resistant, transovarially infected Aedes eggs lie dormant in dambo soils until heavy ENSO/El Niño rainfall (nonlinear 21-day threshold) floods the depressions; primary Aedes (*Ae. mcintoshi*, *Ae. ochraceus*, *Ae. vexans*) mass-hatch within 1–2 days and seed livestock viraemia and abortion storms, secondary Culex and Mansonia surge at ~28–42 days to amplify horizontal spread, and human infection follows as a seasonal epidemic pulse before surviving eggs re-enter dormancy. In the urban analogue, persistent artificial water—storm drains, construction impoundments, uncovered household storage, and organically polluted slaughterhouse water—recreates the water-retaining function of dambos, sustaining a year-round larval habitat without seasonal dormancy; *Culex quinquefasciatus* dominates, a peri-urban cattle bridge (attracting ~3× more mosquitoes) amplifies transmission, and spillover reaches abattoir workers, butchers, and raw-milk consumers year-round, decoupled from rural residence. The behaviour of primary floodwater Aedes in engineered urban habitats remains uncharacterized (dashed banner), the review’s central knowledge gap. Solid arrows denote transmission steps; dashed loop arrows denote the persistence mechanism that closes each cycle.

## 4. Climate Dynamics and Urban Vector Habitats

RVF epizootics are triggered by anomalously heavy, sustained rainfall, itself synchronized to the warm phase of the El Niño–Southern Oscillation (ENSO); since 1950, approximately 90% of documented outbreaks have coincided with El Niño events, when equatorial East Africa receives well-above-normal precipitation [8]. This teleconnection is regionally phased, with outbreaks clustering in East Africa during El Niño and in southern Africa during La Niña [20]. Widespread flooding then triggers mass hatching of dormant, virus-infected *Aedes* eggs within one to two days, followed ~28–42 days later by the secondary *Culex* and *Mansonia* surge that drives the amplification phase responsible for most human infections [17]. Under continued climate change, the frequency and intensity of the extreme-rainfall events that initiate these cycles are widely projected to increase, plausibly accelerating and extending this cascade across endemic tropical zones [10].

Beyond rainfall, a local microhabitat governs transmission: temperatures of 17–35 °C accelerate vector population growth and shorten the viral incubation period [13], while soil water retention and flat topography concentrate larval pools and satellite vegetation indices (NDVI) flag flooding-driven emergence for early warning [8,13]. Critically, irrigation schemes and dams have sparked outbreaks even during droughts by sustaining permanent water—a mechanism directly transferable to urban water infrastructure—yet whether the urban heat island raises or lowers RVFV transmission in already-warm cities remains unstudied [13,21].

These natural determinants are profoundly reconfigured in urban landscapes. Replacing natural soil and vegetation with impervious surfaces alters urban hydrology, and artificial features, storm drains, drainage ditches, and construction impoundments can harbour a substantial share of the resident mosquito population, recreating the water-retaining function of rural dambos beside dense human settlement [21]. In informal peri-urban areas where drainage is absent or overwhelmed, such accumulations persist well beyond individual storms, functioning as year-round larval habitats [10]. Uncovered household water storage in tanks, drums, and containers, ubiquitous where piped supply is intermittent, adds dry-season breeding sites, a pattern extensively documented for dengue vectors across the same regions [11]. The entomological centrepiece of this complex is *Culex quinquefasciatus*, which has become the dominant mosquito of major African cities by exploiting organically polluted stagnant water in drains, pit latrines, and septic tanks, its proliferation propelled by rapid unplanned urbanization [22]. Field trapping at urban Kisumu slaughterhouses confirms that this established secondary amplifier of RVFV can reach very high densities in poorly drained settings, making its unchecked urban expansion a critical, inadequately characterized transmission risk [23].

Yet the primary floodwater vectors of RVFV, *Aedes mcintoshi* and *Ae. ochraceus*, remain ecologically tied to rural low-lying grasslands [24], and their behaviour within urban drainage channels, construction-site impoundments, and modified floodplains has never been examined. Odour-bait surveillance developed for these rural species has not been translated to urban plant communities [25]. Compounding this gap, pyrethroid and multi-insecticide resistance, driven by *kdr* mutations and metabolic detoxification, is now widespread in urban *Culex* populations, undermining the chemical control that anchors most vector-management programmes [22]. Direct urban entomological evidence is nascent and so far negative: at two Kisumu slaughterhouses sampled during an FAO early-warning alert, abundant *Culex* were trapped, yet no pool tested RVFV-antigen-positive, and livestock carried only past exposure (8.5% IgG, no acute IgM cases) [23]. The authors attribute this to the absence of active circulation during the sampling window—regional risk did not extend into the catchment and no acute cases arose—rather than to vector incompetence, with a livestock sample below the disease-freedom threshold further limiting power to exclude acute infection; broader urban infection rates therefore remain unmeasured [23]. Finally, host-choice behaviour concentrates interface risk, because cattle attract roughly three times as many mosquitoes as sheep or goats, magnifying the danger posed by peri-urban cattle markets, trade routes, and informal slaughterhouses [26].

Although *Cx. quinquefasciatus* is emphasized here as the most probable urban amplifier, RVFV is a vectorial generalist, isolated from more than 53 mosquito species across eight genera [8]. Beyond the primary floodwater *Aedes* (*Ae. mcintoshi*, *Ae. ochraceus*, *Ae. vexans*), competent or implicated vectors include other *Culex* (*Cx. pipiens*, *Cx. tritaeniorhynchus*, *Cx. antennatus*, *Cx. theileri*) and *Mansonia* (*Ma. africana*, *Ma. uniformis*) as secondary amplifiers, with *Anopheles* playing a lesser, opportunistic role; notably, *Ae. albopictus* (a principal dengue vector expanding through tropical and temperate cities) is experimentally competent for RVFV [16], marking the clearest overlap with dengue’s *Aedes*-driven transmission system and a plausible urban bridge. This broad, multi-genus range is epidemiologically important: unlike dengue (transmitted almost exclusively by container-breeding *Ae. aegypti* and *Ae. albopictus*), RVFV exploits diverse larval habitats, so urban risk is not confined to household containers and dengue-derived, container-focused control captures only part of the RVFV vector community.

## 5. Evidence of Urban and Peri-Urban RVFV Circulation

The direct empirical evidence for urban and peri-urban RVFV circulation is summarized in Table 1.

The strongest published evidence of RVFV circulation in a peri-urban livestock setting comes from a previous study that applied force-of-infection (FOI) catalytic modelling to age-stratified, cross-sectional serosurveillance data collected across peri-urban, agro-pastoral, and pastoral zones in northern Tanzania [12]. During the inter-epidemic period (IEP) from 2009 to 2015, when no RVF cases were officially reported, estimated annual RVFV incidence was 96 per 10,000 cattle, 79 per 10,000 sheep, and 39 per 10,000 goats (Table 1). Village-level FOI heterogeneity showed no spatial autocorrelation (Moran’s I = 0.07; *p* = 0.14). This indicates that transmission intensity was governed by local hydrology and land-use patterns rather than landscape-level factors, a finding with direct implications for targeted urban risk mapping. Critically, the vast majority of livestock remained serologically naïve throughout the study period, signalling high population susceptibility and substantial epidemic potential once the next flooding event occurs [12]. At the continental scale, a previous study estimated a pooled animal RVFV prevalence of 9.3% (95% CI: 8.1–10.6%) across 134,274 animals. Prevalence was highest in Eastern Africa (10.6%) and Southern Africa (10.9%), and wildlife–livestock habitat overlap was documented in 6 of 9 outbreak-reporting countries, a convergence that amplifies IEP transmission risk in the peri-urban interface zones now typical of rapidly growing African cities [3].

Building on these livestock findings, a previous study documented a human RVFV seroprevalence of 8.2% (95% CI: 6.2–10.9%) in the same northern Tanzanian communities (the strongest published evidence of IEP livestock-to-human spillover in a peri-urban setting) [12]. Village-level livestock FOI, together with age and sex, accounted for more than 50% of the between-village variation in human seropositivity (proportional change in variance: 57%). Raw milk consumption was independently and strongly associated with seropositivity (OR: 3.67; 95% CrI: 1.58–8.14), even after controlling for direct livestock contact. Because milk is routinely traded over long distances to urban consumers who have no pastoral exposure, this effectively decouples RVFV risk from rural residence [12]. At the occupational level, another study confirmed through meta-analysis that butchering (OR: 3.7), milking (OR: 5.0), and handling aborted material (OR: 3.65) are the highest-risk urban exposure categories [1]. Diagnostic masking further obscures the true urban burden. Another study showed that the clinical picture of RVF overlaps substantially with yellow fever, viral hepatitis, chikungunya, malaria, dengue fever, and typhoid, so RVF is chronically under-considered in urban differential diagnosis [7]. The annual Hajj pilgrimage adds a critical convergence point, concentrating pilgrims alongside livestock imported from endemic zones in East Africa and the Horn of Africa for large-scale ritual slaughter [5,29].

Vector evidence from Sub-Saharan African urban settings reinforces and extends this human exposure picture. *Culex quinquefasciatus*, the dominant mosquito of African cities, proliferates in the polluted stagnant water created by rapid urbanization and is a confirmed secondary RVFV vector capable of sustaining inter-epidemic amplification [8,22]. A previous study in Kenya showed, in an endemic setting, that cattle consistently attract about three times more mosquitoes than sheep or goats. This means peri-urban dairy cattle concentrate vector biting pressure and create an amplified transmission bridge to surrounding human populations [26]. Despite this mechanistic evidence, inter-epidemic peri-urban entomological surveillance is essentially absent from the published literature. Another study found that only 42 of 285 included reports (15%) incorporated any vector testing and that passive, outbreak-reactive systems systematically fail to capture sub-clinical IEP transmission [1]. In addition, a study demonstrated that zoonotic arboviruses including RVFV are present well before human illness becomes detectable through routine surveillance, providing the ecological rationale for proactive, risk-based entomological monitoring in urban RVFV interface zones [13]. That same work proposed syndromic surveillance integrated with sentinel-herd monitoring and satellite-based early warning as the most feasible human-facing detection framework, yet this architecture remains unimplemented in any city [7].

## 6. Socio-Demographic and Land-Use Drivers of Urban RVF Risk

Identifying who bears the greatest exposure is foundational to any urban control strategy, yet the occupational epidemiology of RVF has been assembled almost entirely from rural cohorts. The highest-risk occupations involve direct contact with livestock or carcasses: pastoralists, farmers, herders, abattoir and slaughterhouse workers, butchers, veterinarians, and laboratory personnel. Male herders and people who repeatedly handle aborted material, milk, skin, or slaughtered animals are the groups most consistently identified as exposed [7,9,28]. Meta-analysis quantifies this gradient: regular animal exposure raises the odds of seropositivity by roughly half (pooled OR = 1.46), rising to 3.7 for butchering, 3.4 for sheltering livestock, and 5.0 for milking [1]. A frequently overlooked group is women preparing food at home, among whom higher seroprevalence has been linked to routine handling of raw meat and animal products [1,5]. Critically, no occupational study has been conducted exclusively in an urban or peri-urban setting. Pooled abattoir estimates also conflate slaughterhouse size, species slaughtered, and personal protective equipment as uncontrolled confounders, while poorer and less-educated workers remain consistently overrepresented [1].

These exposures are shaped by an expanding peri-urban livestock economy. Rapid urbanization is drawing animal husbandry into the fringes of tropical cities, where small-scale dairying and backyard keeping of ruminants and poultry persist as food-security strategies within informal settlements (the same peri-urban zone in which measurable RVFV circulation has been documented) [12]. One study frames this system as a livestock food value chain, along which pathogen risk passes through successive nodes: feed inputs, production, transport, the abattoir, processing, and the consumer. Each node is a potential amplification point in dense urban space [5]. The consumer node is not hypothetical. In the 2018–2019 Mayotte outbreak (142 confirmed cases), 67.5% of investigated patients reported direct contact with livestock or their fluids, including raw or curdled milk, and raw- and curdled-milk sales were subsequently banned [27]. Live animal markets compound the hazard by gathering stock of diverse endemic origin within the city, and the movement of viremic animals remains the main driver of geographic spread, such as when the 2007 Sudan outbreak reached Khartoum via animals moved to market [9,28]. Yet the peri-urban dairy sector remains almost absent from RVF risk-factor and vaccination-coverage data [1].

Underlying these economic structures are demographic and land-use shifts that push risk toward the urban poor. Rural-to-urban migration channels new arrivals into low-lying, flood-prone peri-urban zones (the niche most conducive to vector emergence) while informal urban irrigation and agriculture maintain waterlogged microhabitats that support secondary vector breeding beyond the rainy season [10]. Spatially, overlaying livestock migratory routes onto vector-habitat suitability has delineated RVF risk zones, showing that animal movement and ecological conditions jointly determine where exposure concentrates [30]. Vulnerability is further patterned by sex and comorbidity. In Sudan, males aged 15–29 were overrepresented among severe cases, and women were affected mainly through domestic handling of animal products; HIV co-infection has been linked to the encephalitic form and to fatal outcomes, particularly in high-prevalence Sub-Saharan African cities [1]. As settlements advance into formerly pastoral and wetland landscapes, they create new human–livestock–wildlife interfaces that favour cross-species spillover, given the documented overlap of RVFV across domestic and wild hosts [3]. Because RVFV circulates across domestic and wild hosts, wild ungulates can serve as additional amplifying or sentinel hosts, so peri-urban encroachment into their habitats widens the wildlife–livestock interface seeding urban spillover [3]. Overarching all of this is a structural One Health blind spot: because RVF is still seen as a pastoral disease, urban health systems are neither sensitized to include it in differential diagnosis nor equipped to report it [1,5].

## 7. Future Burden Projections

Predictive frameworks for RVFV rest almost entirely on ENSO-linked rainfall anomalies and rural landscape ecology (dambo flooding, irrigation schemes, and agro-climatic zonation), and no operational model has been developed for urban or peri-urban settings [31]. The Rift Valley Fever Monitor combines Potential Epizootic Area Masks (PEAMs) with NDVI anomalies to anticipate outbreaks in pastoral landscapes, but it does not account for urban land-cover change, impervious-surface hydrology, or peri-urban vector dynamics [8]. Remote sensing tools likewise support climate-informed early-warning systems for vector-borne diseases, with RVF among the pathogens cited. Yet their field applications centre on rural schistosomiasis and trypanosomiasis landscapes and have not been extended to urbanizing settings [32]. A comprehensive epidemiological synthesis consolidating 1104 RVFV transmission events across 39 countries (1999–2021) identifies climate variability and livestock contact as the primary drivers, yet it projects no future burden under Shared Socioeconomic Pathway (SSP) or Representative Concentration Pathway (RCP) scenarios [1]. The coupled *Aedes*–*Culex*–livestock model, validated against the 2006–2007 Kenya epizootic using satellite rainfall, similarly remains disconnected from urban growth trajectories [17]. Critically, as far as we know, no published study has coupled CMIP6/SSP climate projections with urban-expansion models to estimate future RVFV burden in tropical cities (the central gap this review identifies).

This modelling gap is especially consequential given the pace of African urbanization. Sub-Saharan Africa’s population is projected to nearly double, from about 1.15 billion in 2022 to 2.1 billion by 2050 (the fastest growth of any world region and more than half of the expected global increase) [33]. Much of this expansion will be urban and peri-urban. Across Africa, urban land has consistently grown faster than population, producing low-density sprawl that consumes surrounding agricultural and natural land, while informal settlements with inadequate water and drainage infrastructure multiply at the city fringe [34]. These fringe zones increasingly overlap RVFV Potential Epizootic Area Masks around cities in endemic countries [1,8]. The serological evidence sharpens this concern: a previous study shows that the vast majority of cattle, goats, and sheep across northern Tanzania remain unexposed and therefore fully susceptible, making a large peri-urban outbreak plausible at the next major flood [12]. Compounding this, uncontrolled livestock corridors allow viremic animals to enter urban slaughter systems undetected [5,9].

Translating this evidence into actionable projections requires both priority-setting and a coherent modelling architecture. Based on documented outbreak geography, endemic countries with recorded RVF histories (Kenya, Tanzania, Sudan, Madagascar, and Saudi Arabia) contain cities where converging climatic, demographic, and ecological pressures create inferred high-risk zones [9,23]. Within these systems, peri-urban residents in flood-prone informal settlements, livestock traders, abattoir workers, and raw-milk consumers constitute the highest-risk subpopulations, all set to grow as urbanization accelerates. This review proposes coupling the RVFV transmission architecture with CMIP6 climate projections under SSP2-4.5 and SSP5-8.5 and spatially explicit urban-expansion datasets (e.g., LandScan, GRUMP) to generate city-level burden estimates [17]. A recent study offers a methodological benchmark, integrating climatic, socio-demographic, and healthcare determinants through explainable AI and Bayesian spatio-temporal modelling to produce district-level dengue early warnings across Bangladesh [35]. Priority metrics should include the urban RVFV force of infection, peri-urban vaccination coverage, and the relative contribution of slaughterhouse, vector, and food-chain pathways to the human burden.

## 8. Mitigation and Adaptation

Operational early warning for RVF already exists, but it was calibrated for rural landscapes. The Rift Valley Fever Monitor, maintained by the USDA and NASA and updated monthly, uses satellite NDVI anomalies to identify areas where flooding favours vector emergence. It retrospectively reconstructed outbreak-prone zones from 1981 to 1998 and prospectively signalled the East African (2006–2007), Sudanese (2007), and southern African (2008–2010) epizootics, allowing stakeholders to begin surveillance and mitigation weeks to months in advance [8]. Two structural constraints limit its urban usefulness. First, climate signals detect favourable weather conditions but cannot account for pre-existing herd immunity, which strongly influences whether an outbreak actually occurs despite high-risk weather (a fundamental confound the system cannot resolve) [17]. Second, its Potential Epizootic Area Masks and NDVI thresholds are calibrated to rural dambo hydrology; impervious surfaces, engineered drainage networks, and household water storage create hydrological signatures that existing algorithms cannot capture. A priority adaptation is to combine satellite flood-extent and surface-water mapping with NDVI products to derive urban-specific risk thresholds, extending the environmental monitoring framework already proposed for RVF and related vector-borne diseases [32].

Where early warning identifies rising risk, sustained integrated vector management can suppress transmission. The most thoroughly documented urban–peri-urban example is the 18-year programme (2000–2018) implemented in the Jazan region of Saudi Arabia. Targeting outdoor habitats specifically, the programme combined conventional insecticide application by air and ground, microbial larviciding with *Bacillus thuringiensis* var. *israelensis*, drainage and soil-infilling of water swamps, entomological surveillance, sustained vaccination campaigns, and regular sentinel-herd monitoring with targeted sero-surveillance during rainy seasons [36]. The outcomes were striking. RVFV infection rates in *Culex* mosquitoes fell from 0.045 per 1000 in 2014 to zero across 2015–2018, and RVFV-specific IgM seroprevalence in local herds dropped from 12.3% (95% CI: 6.8–17.8) in 2000 to 0.10% (95% CI: 0.01–0.2) in 2017, with no human or animal cases since [36]. The same outdoor-targeted measures also substantially reduced malaria (from 2756 cases in 2000 to 15 in 2014), demonstrating the multiplier value of integrated control in settled landscapes. The Saudi programme therefore provides an evidence-based template that other endemic countries could adapt to urban and peri-urban contexts.

Transplanting this model to tropical cities faces obstacles the Jazan programme largely avoided. Widespread pyrethroid resistance in urban *Culex* populations undermines standard adulticide campaigns, requiring rotation to non-pyrethroid agents such as the carbamate bendiocarb and the organophosphate pirimiphos-methyl [11]. Egypt illustrates the risks of an incomplete approach: national vector control policy relies mainly on chemical insecticides, without environmental management, biological control, or health education. The absence of cross-sectoral coordination and community involvement has been explicitly identified as a programmatic gap (structural deficits shared by many rapidly urbanizing tropical cities) [37]. Because primary *Aedes* vectors emerge within one to two days of flooding, larviciding must be timed almost immediately, which makes satellite flood mapping a still-underused tool for prioritizing urban application zones. A promising peri-urban adaptation uses insecticide-treated cattle in enclosure traps; by exploiting cattle’s high attractancy, these act simultaneously as surveillance sentinels and vector sinks in dairy zones [26]. Community-based waste management and drainage clearing remain essential non-chemical components of IVM, but they depend on the sustained intersectoral engagement that most resource-constrained settings have yet to institutionalize [10].

Targeting the amplifying host directly, livestock vaccination remains the most evidence-based preventive intervention against RVF [17,38]. Established platforms carry important trade-offs. The live-attenuated Smithburn vaccine provides durable single-dose immunity but is teratogenic and abortifacient in pregnant animals and carries a risk of reversion to virulence; inactivated vaccines are safe in all physiological states but require booster doses within three to four weeks; and Clone 13, with its single-dose safety profile, is now registered in South Africa, Namibia, Botswana, Zambia, and Mozambique [38,39]. Next-generation candidates extend this landscape. ChAdOx1-GnGc confers complete protection in sheep, goats, and cattle after a single dose; NDV-vectored vaccines are advantageous because sheep and cattle are not natural NDV hosts and so are unlikely to carry pre-existing anti-vector immunity; and nonspreading single-cycle replicons achieve sterile immunity in lambs [38]. Pre-emptive or early-warning-triggered inter-epidemic vaccination of cattle (the dominant viral amplifier) averts substantially more cases than reactive campaigns [17]. Cold-chain requirements, thermostability, cost, and smallholder affordability remain serious barriers in tropical peri-urban settings [2], and livestock coverage data from this sector are entirely absent from the literature.

No single intervention is enough; durable protection requires integrating early warning, vector management, and vaccination within a coordinated One Health architecture that jointly covers veterinary sentinel surveillance, climate-based forecasting, and human syndromic monitoring (an approach repeatedly advocated yet weakly implemented across endemic settings) [1,40]. Operationally, this maps onto the WHO–FAO–WOAH tripartite framework, sharing surveillance data across veterinary, human-health, and environmental sectors; urban slaughterhouses are an efficient node, where the vector-trapping and livestock-serology framework piloted in Kisumu delivers combined entomological and livestock surveillance where animals, vectors, and people converge [23]. The operational blueprint already exists in peri-urban outline: regular cross-sectional serosurveillance of dairy cattle, combined with force-of-infection modelling, can detect inter-epidemic RVFV circulation before clinical cases appear, as shown in northern Tanzania [12]. Adding RVF diagnostic panels to fever-clinic testing triggered by flooding events (building on established malaria and dengue surveillance infrastructure) would improve case detection, while cellphone-based livestock-abortion reporting by peri-urban farmers could shorten the animal-to-public-health response lag. Prevention can also be engineered through the built environment: replacing open household water containers with piped supply, improving solid waste management, and screening housing openings all reduce *Aedes* contact, as proven over decades of urban dengue control [41]. By extension, zoning rules that separate live-animal markets and abattoirs from dense residential areas address the urban RVFV spillover pathways this review has documented. Embedding RVF within urban planning and One Health systems, rather than treating it as a purely pastoral concern, is the central adaptation this review advocates.

## 9. Discussion

This review repositions Rift Valley fever (RVF) from a mainly rural-pastoral disease to a plausible emerging threat at the tropical urban–peri-urban interface, arising from the interaction of two macro-forces rather than either one alone (Figure 1). Climate change is intensifying the ENSO-linked extreme-rainfall events that drive vector emergence [8], while unplanned urban growth pushes dense settlement onto flood-prone land. This convergence already shapes arboviral risk elsewhere. In Brazil, both extreme rainfall and extreme drought raised dengue risk, with the drought-related increase concentrated in highly urbanized areas where interrupted water supply drives household water storage [42]. An analogous chain is plausible for RVF: urban standing water recreates dambo-like larval habitat beside dense habitation (Figure 2), where *Culex quinquefasciatus* and the peri-urban cattle host-choice bias form an efficient amplification bridge to people (Figure 1). The single direct empirical anchor—inter-epidemic RVFV circulation in peri-urban northern Tanzania, with raw-milk-associated spillover decoupling risk from rural residence [12]—confirms the pathway is real, yet remains as one study (Table 1); the urban threat still rests largely on inference.

That inferential foundation exposes several interlocking gaps. The behaviour of the primary floodwater vectors, *Aedes mcintoshi* and *Aedes ochraceus*, within urban drainage channels, construction impoundments, and engineered floodplains has never been characterized. No urban RVFV infection-rate data exist: the few abattoir screening efforts have not detected virus-positive mosquito pools, and none have estimated infection rates across a broader urban mosquito community. The urban heat-island effect on RVFV transmission is likewise unstudied, even though this phenomenon demonstrably raises dengue incidence in hotter, low-vegetation city zones by accelerating vector development, blood-feeding, and viral replication [43] (a plausible but untested amplifier for *Culex*-borne RVFV). No occupational or consumer exposure study has been conducted exclusively in an urban or peri-urban setting; pooled abattoir estimates conflate facility size, species handled, and protective equipment as confounders [1]. The peri-urban dairy and food-value-chain sector is nearly absent from RVF risk-factor and vaccination-coverage data, despite feeding the informal, low-compliance value chains through which raw milk reaches the consumer [44]. Critically, no predictive model couples RVFV climate projections with urban-expansion datasets, so no city-level burden estimate exists. These gaps are compounded by this review’s own constraint: as a narrative synthesis of a sparse urban literature, it necessarily leans on rural ecology and analogous dengue evidence.

Translating this evidence into action need not await complete data. Operational early-warning systems can integrate satellite flood-extent and surface-water mapping with vegetation indices [45] to derive urban-specific risk thresholds beyond rural dambo calibration. The 18-year integrated vector-management programme that reversed RVF recurrence in the Jazan region of Saudi Arabia offers a transferable template [36], though urban deployment must contend with target-site resistance documented in urban *Culex*, widespread *kdr* (pyrethroid) and emerging *ace-1* (organophosphate/carbamate) mutations [46,47], favouring resistance-monitored rotation among distinct modes of action and larval source management timed to flood mapping. Livestock-based One Health tools are especially apposite where cattle amplify the virus: insecticide-treated cattle kill the mosquitoes that feed on them, suppressing vectors at the amplification hub [48]. For RVF the insecticidal effect is decisive, since diverting bites toward cattle would instead feed amplification, while treated enclosures could double as sentinels. Because cattle drive amplification, pre-emptive inter-epidemic livestock vaccination should be prioritized, using established veterinary vaccines such as Clone 13 alongside next-generation dual-use candidates (notably the single-dose, thermostable ChAdOx1 RVF vaccine that is efficacious in livestock and immunogenic in humans) while resolving smallholder affordability and cold-chain access [49]. Prevention can be further engineered through the built environment: piped supply replacing open storage, solid-waste management, housing screening, and zoning that separates live-animal markets and abattoirs from dense residential areas. Ultimately, dismantling this structural neglect requires embedding RVF within urban planning and integrated surveillance uniting veterinary sentinels, climate forecasting, and human syndromic monitoring, rather than treating it as a solely pastoral concern.

Rift Valley fever in the context of other emerging mosquito-borne zoonoses: Set against other emerging mosquito-borne zoonoses, RVF’s distinctiveness is clearer. Jamestown Canyon virus (JCV) (an orthobunyavirus maintained in a deer reservoir and transmitted by diverse mosquitoes across the northern United States and Canada) offers an instructive temperate-zone counterpart (Table 2) [50]. Both are climate-sensitive zoonoses of rising recognition whose illness ranges from mild febrile disease to neuroinvasive or severe forms, yet they diverge sharply in scale and consequence: JCV remains sporadic and low-incidence with no livestock or economic impact, whereas RVFV drives explosive epizootic–epidemic outbreaks, heavy livestock losses, and severe or hemorrhagic disease and, unlike JCV, also spreads through contact with animal blood, tissue, aborted material, aerosols, and raw milk. This contrast underscores that RVF’s livestock amplification, broad vector range, and multiple transmission routes make it a fundamentally different, and greater, public-health and economic threat.

## 10. Strengths and Limitations

This review has several strengths. To our knowledge, it is the first to bring together, under a single One Health lens, what is known about Rift Valley fever in urban and peri-urban settings across mosquitoes, livestock, and people and to frame that evidence within the twin pressures of climate change and rapid tropical urbanization. By drawing on a broad, cross-disciplinary body of work spanning Sub-Saharan Africa, the Arabian Peninsula, and the Indian Ocean islands, it connects fields that are rarely considered together, and it translates scattered findings into a clear, prioritized agenda for future research and policy. These strengths are balanced by important limitations. Because dedicated urban studies remain scarce, much of the analysis relies on inference, extrapolating from rural transmission ecology and from better-studied urban arboviruses such as dengue. Several operational recommendations (such as container source reduction, housing screening, and dengue-style early warning) are therefore adapted from dengue; because RVFV differs fundamentally in its broad multi-genus vector range, its amplification in a livestock reservoir, and its transmission through animal contact, aerosols, and raw milk, these should be regarded as starting templates requiring RVF-specific validation rather than direct transfer. These analogies are biologically plausible but unproven for Rift Valley fever. The direct urban evidence rests heavily on a single field study, and the absence of urban vector-infection, exposure, and modelling data means several conclusions are hypothesis-generating rather than definitive. As a narrative rather than systematic review, it did not apply a formal protocol, quantitative pooling, or standardized quality appraisal, and its restriction to English-language sources may have missed relevant regional literature. These constraints reflect genuine gaps in the evidence base as much as the review’s design, and they underscore why the targeted studies proposed here are urgently needed.

## 11. Future Directions

Future research should advance along five priorities. First, proactive inter-epidemic surveillance in peri-urban zones is needed, pairing cross-sectional dairy-cattle serosurveillance with force-of-infection modelling to detect circulation before clinical cases emerge, adapting the northern Tanzania blueprint [12]. Second, urban vector ecology must be measured rather than inferred, including infection rates across the urban mosquito community, primary-vector behaviour in engineered habitats, and the influence of urban heat on vector development and viral incubation [21]. Third, coupled climate–urban models are required to link RVFV transmission dynamics to intermediate- and high-emission scenarios (SSP2-4.5 and SSP5-8.5) and spatially explicit urban-expansion data, generating the first city-level burden estimates; multi-model frameworks for mosquito-borne disease [51] and Bayesian spatiotemporal dengue models [42] offer directly transferable templates. Fourth, urban-specific occupational and consumer exposure studies, particularly along the raw-milk and food value chain, should replace extrapolation from rural cohorts. Finally, research should concentrate on high-risk geographies (cities in Kenya, Tanzania, Sudan, Madagascar, and Saudi Arabia within or adjacent to recognized epizootic areas) where ecological suitability and rapid urbanization most sharply intersect.

## 12. Conclusions

Rift Valley fever has been framed for decades as a rural, pastoral disease, but the convergence of intensifying climate variability and rapid, unplanned tropical urbanization is eroding that assumption. This review has argued that urban infrastructure can recreate dambo-like standing water in which *Culex quinquefasciatus* and peri-urban cattle plausibly form an amplification bridge to dense human populations. Yet the urban threat still rests largely on inference: it is anchored by a single peri-urban serosurvey and a handful of slaughterhouse studies, while the behaviour of primary floodwater vectors in engineered habitats, urban infection rates, urban-heat effects, city-specific exposure pathways, and coupled climate–urban burden models all remain unexamined. Closing these gaps will require proactive inter-epidemic surveillance, direct measurement of urban vector ecology, and city-level modelling, together with the embedding of RVF within urban planning and integrated One Health systems. Treating RVF as a solely pastoral concern risks leaving tropical cities unprepared for a threat already taking shape.

## Figures and Tables

**Table 1:** Evidence of urban and peri-urban Rift Valley fever virus (RVFV) circulation in humans, livestock, and vectors.

Study/Setting	Population	Period	Key Metric	Value (95% CI)
de Glanville et al. [12], 2022; Northern Tanzania (peri-urban, agro-pastoral & pastoral gradient)	Humans	Inter-epidemic (2009–2015)	Seroprevalence	8.2% (95% CI 6.2–10.9)
	Humans	IEP	Raw-milk consumption (independent risk factor for seropositivity)	OR 3.67 (95% CrI 1.58–8.14)
	Humans	IEP	Between-village variation in human seropositivity explained by livestock FOI + age + sex	PCV 57%
	Cattle/sheep/goats	IEP	Estimated annual force of infection (per 10,000)	96 (81–113)/79 (62–98)/39 (28–52)
	Livestock (village-level)	IEP	Spatial autocorrelation of village FOI	Moran’s I = 0.07 (*p* = 0.14; none)
Madut et al. [19], 2025; Moshi, northern Tanzania (peri-urban dairy-cattle outbreak, 2018; urban-adjacent)	Humans (*n* = 656 hospitalized febrile)	2016–2019 (outbreak 2018)	RVFV RT-PCR positivity; residence	4/656 (0.6%), incl. 1 death; all resided in/near urban areas
	Dairy cattle	2018	RVFV-positive (abortion surveillance)	14
	Human/cattle sequences	2018	Lineage	Mainly B (+1 E)
Ebogo-Belobo et al. [3], 2023; Continental meta-analysis (Africa)	Humans	Pooled (epidemic + IEP)	Pooled seroprevalence	7.8% (95% CI 6.2–9.6)
	Animals (*n* = 134,274)	Pooled	Pooled seroprevalence	9.3% (95% CI 8.1–10.6)
	Animals	Pooled (regional subgroups)	Highest regional seroprevalence	S. Africa 10.9%; E. Africa 10.6%
	Wildlife–livestock	-	Domestic–wild animal overlap among outbreak-reporting countries	6 of 9 countries
Youssouf et al., 2020 [27]; Mayotte (2018–2019 outbreak; urban/island setting)	Humans	Epidemic	Confirmed cases	142
	Humans	Epidemic	Patients reporting exposure to animals or their biological fluid	73%
	Humans (126/142 investigated)	Epidemic	Direct contact with livestock (care, treatment, slaughter)	67.5%
Gerken et al., 2024 [23]; Kisumu, Kenya (urban slaughterhouses)	Mosquitoes (Culex-dominated)	IEP	RVFV-positive mosquito pools (VecTOR antigen test)	None (0 positive)
	Livestock (*n* = 923)	IEP	IgG seroprevalence (prior infection); IgM (recent)	8.5% IgG (78/923); no IgM positives
Tchouassi et al., 2016 [26]; Kenya (endemic; peri-urban relevance)	Mosquitoes/livestock hosts	-	Relative mosquito attraction, cattle vs. sheep	≈3-fold higher for cattle (IRR 2.9, 95% CI 1.24–7.96)
Himeidan, 2016 [28]; Sudan (2007 outbreak)	Humans/livestock	Epidemic	Urban spread via movement of animals to markets	Spread from White Nile to Khartoum (+Gezira, Sennar, Kassala)

**Abbreviations:** IEP, inter-epidemic period; FOI, force of infection; OR, odds ratio; CI, confidence interval; CrI, credible interval; PCV, proportional change in variance; IRR, incidence rate ratio. **Notes:** This table consolidates the direct empirical evidence for urban and peri-urban RVFV circulation. The northern Tanzanian study (de Glanville et al., 2022) [12] remains the only published account measuring inter-epidemic circulation across a gradient that includes a peri-urban zone, and the Kisumu study (Gerken et al., 2024) [23] provides the only direct urban slaughterhouse vector and livestock data. No occupational or consumer exposure study has been conducted exclusively in an urban or peri-urban setting; in the broader systematic review, only 42 of 285 reports (15%) incorporated any vector testing (Gerken et al., 2022) [1], underscoring how thin this evidence base remains. Madut et al. (2025) [19] is the first genomically confirmed human RVFV report from an urban-adjacent setting, while Juma et al. (2026) [18] adds continental context, documenting geographically structured, actively diversifying lineages.

**Table 2:** Comparison of Rift Valley fever and Jamestown Canyon virus as emerging mosquito-borne zoonoses.

Feature	Rift Valley Fever (RVFV)	Jamestown Canyon Virus (JCV) [50]
**Virus family/genus**	*Phenuiviridae*; *Phlebovirus*	*Peribunyaviridae*; *Orthobunyavirus* (California serogroup)
**Reservoir/amplifier**	Domestic ruminants (sheep, cattle, goats)	White-tailed deer, other cervids
**Principal vectors**	Broad: floodwater *Aedes* (primary); *Culex*, *Mansonia* (secondary); >53 species, 8 genera	Diverse mosquito species
**Geographic distribution**	Sub-Saharan Africa, Egypt, Arabian Peninsula, Indian Ocean islands	Upper Midwest and Northeastern USA, and Canada
**Epidemiology**	Explosive ENSO-linked epizootic/epidemic outbreaks; 39 countries; major economic impact	Sporadic, low incidence (27 US cases, 7 states, 2023); no livestock/economic impact
**Clinical spectrum**	Mostly self-limiting febrile; ~1–2% severe (hepatitis, retinitis, encephalitis, haemorrhagic fever)	Often asymptomatic/mild febrile; may progress to meningitis/encephalitis
**Reported severity**	Case-fatality ~27.5% (confirmed cases)	74% neuroinvasive, 93% hospitalized, 11% deaths (2023)
**Transmission routes**	Mosquito bite + contact with blood/tissue/aborted material, aerosol, raw milk	Mosquito bite only

## Data Availability

No new data were created or analyzed in this study. Data sharing is not applicable to this article.
